# Parents’ Perspectives on the Theoretical Domains Framework Elements Needed in a Pediatric Health Behavior App: A Crowdsourced Social Validity Study

**DOI:** 10.2196/mhealth.9808

**Published:** 2018-12-21

**Authors:** Christopher C Cushing, David A Fedele, Erin E Brannon, Tiffany Kichline

**Affiliations:** 1 Clinical Child Psychology Program University of Kansas Lawrence, KS United States; 2 Schiefelbusch Institute for Life Span Studies University of Kansas Lawrence, KS United States; 3 Department of Clinical & Health Psychology University of Florida Gainesville, FL United States; 4 Dayton Children's Hospital Dayton, OH United States

**Keywords:** mHealth, adolescent, children, parent, stakeholder, consumer preference

## Abstract

**Background:**

Most pediatric studies do not include parent stakeholders in the design of the intervention itself and many pediatric mobile health (mHealth) interventions are not meaningfully disseminated after the trial period ends. Consequently, the consumer desire for mobile apps targeting pediatric health behavior is likely to be met by commercial products that are not based in theory or evidence and may not take stakeholder preferences into account.

**Objective:**

The aim was to assess parent preference for mobile app features that map onto specific Theoretical Domains Framework (TDF) elements.

**Methods:**

This study was a crowdsourced social validity study of 183 parents who were asked to rate their preferences for mobile app features that correspond to elements of the TDF. The TDF organizes a large number of theoretical models and constructs into three components: (1) capability, (2) motivation, and (3) opportunity. Parents of children were recruited through Amazon Mechanical Turk.

**Results:**

The majority of participants were Caucasian and mean age was 36.9 (SD 8.0) years. Results revealed broad acceptability of communication, motivation, and opportunity domains. However, the degree to which each domain was valued varied within behavioral category. Parents demonstrated a preference for increasing procedural knowledge for physical activity and diet behaviors over sleep (*F*_2,545_=5.18, *P*=.006). Similarly, parents valued self-monitoring as more important for physical activity than sleep (*F*_2,546_=4.04, *P*=.02). When asked about the value of features to help children develop skills, parents preferred those features for dietary behavior over sleep (*F*_2,546_=3.57, *P*=.03). Parents perceived that goal-setting features would be most useful for physical activity over sleep and diet (*F*_2,545_=5.30, *P*=.005). Incentive features within the app were seen as most useful for physical activity over sleep (*F*_2,546_=4.34, *P*=.01).

**Conclusions:**

This study presents a low-cost strategy for involving a large number of stakeholders in the discussion of how health behavior theory should be applied in a mHealth intervention. Our approach is innovative in that it took a scientific framework (ie, TDF) and made it digestible to parents so that they could then provide their opinions about features that might appear in a future app. Our survey items discriminated between various health behaviors allowing stakeholders to communicate the different health behaviors that they would like a TDF feature to change. Moreover, we were able to develop a set of consumer opinions about features that were directly linked to elements of the TDF.

## Introduction

Mobile health (mHealth) has the potential to extend the reach and dose of clinical care and expand the kinds of intervention that can be provided to youth (ie, those younger than 18 years) at the point of care [1]. mHealth interventions can be effective at improving youth health outcomes, especially when a parent is an active recipient of the intervention [2]. This finding is consistent with evidence from face-to-face health promotion and prevention interventions that indicate that multisystemic interventions are more effective at improving youth health than those that solely target the individual [3].

There is conceptual agreement that the first phase of digital intervention development should be both grounded in theory and include stakeholders in the process [4]. However, parent preferences and opinions about intervention components are commonly missing from mHealth interventions because stakeholders are not typically included in the development phase of the applications [2]. Moreover, widely available digital health products are not meaningfully based on theory or informed by published evidence [5-7]. The fact that there is a large and vibrant marketplace of digital health tools, even if not evidence-based, suggests that currently available digital health solutions do tap into at least some perceived stakeholder need. If true, this conclusion suggests that research teams should look early and frequently for stakeholder opinions about the design of digital health interventions. One way to ensure that interventions are both based in theory and involve stakeholders is to use a theoretical framework to inform feedback from the stakeholders themselves.

Stakeholder engagement in intervention design can be conceptualized as part of a larger social validity framework [8]. Social validity refers to the degree to which a given intervention is (1) important to society; (2) involves treatment that is acceptable to consumers in terms of cost, ethical considerations, and practicality; and (3) results in treatment outcomes that are acceptable to consumers [9]. Crowdsourcing platforms and the advancement of population screening approaches present an externally valid opportunity to interact with stakeholders [10]. This study used social validity methodology to engage parent stakeholders in a crowdsourced study of stakeholder preferences for mobile app features that correspond to evidence-based behavior change strategies.

To empirically evaluate stakeholder perceptions of intervention elements they might encounter in mobile apps, it is imperative that there is a shared operational definition of the behavioral strategies that investigators would use to change health behavior [11]. Moreover, there is relatively good consensus that interventions should be based on a guiding theory rather than disconnected techniques [1,12]. Incorporating a full-featured theoretical framework, such as the Theoretical Domains Framework (TDF; see Cane et al [13] for review), allows interventionists to make falsifiable predictions about the performance of an intervention. The TDF has been proposed as a system for defining active theoretical elements within larger studies [12,13]. It organizes a large number of theoretical models and constructs into 12 independent domains which can be further aggregated into three components from the behavior change wheel posited by Michie et al [14]: (1) capability, (2) motivation, and (3) opportunity. Capability refers to both one’s physical ability to perform a behavior and also to psychological factors such as knowledge, cognitive capacity, self-regulation, and mental skill. Motivation is subdivided into two elements: automatic and active. The automatic element of motivation involves noncognitive influencers of behavior such as reinforcement, emotion, and habit. The active element of motivation involves more cognitive decision-making processes. Opportunity represents external influences on behavior that facilitate or prompt a behavior via either environmental or social means.

Although theoretical frameworks are useful for researchers to design interventions, they can be particularly challenging to communicate to stakeholders due in part to the discipline-specific knowledge required to understand the definitions and predictions of these frameworks. Within pediatric mHealth interventions, there is a potential that developing a shared language could help parent stakeholders involved in intervention to fully communicate their preference for which behavior change techniques should be included in the final intervention.

Therefore, this study aimed to:

Distill elements of the TDF into features that might be included in a mobile app (make translatable for parents);
Quantify parent stakeholder preferences for elements of the TDF in mHealth interventions by asking them to consider specific features that would be consistent with each TDF element; and
Quantify how parents are currently using mHealth to manage their children’s health.

## Methods

### Recruitment

Participants were parents of children recruited via the Amazon MTurk platform between July and September 2016. We posted an advertisement using a requester account on MTurk and advertised our survey as a project soliciting “Opinions about smartphone apps.” The advertisement noted that we estimated the Human Intelligence Task (HIT) to take 1 hour to complete and that valid responses would receive US $1.66 in compensation. All recruitment procedures and materials were approved by the Institutional Review Board at the University of Kansas (STUDY00003083).

### Study Eligibility

Mechanical Turk (MTurk) is an online marketplace where workers can receive compensation for completing a range of HITs such as completing questionnaires. We required that participants have a Master certification on the MTurk platform (ie, complete at least 1000 tasks and maintain at least 99% approval). This was done to ensure a participant pool who was likely to provide high-quality data and reduce data screening burden. Participants were also required to be the parent of at least one child younger than 18 years. To assess for this eligibility criterion, we followed procedures similar to Schleider and Weisz [10]. Specifically, we included a six-question screener that asked interested participants to self-report their sleep, physical activity, and dietary behaviors, as well as whether they had a child younger than the age of 18. All items other than child age were distraction questions and were not used for eligibility. If the answer to the question about parental status was affirmative, participants were allowed to complete the rest of the survey.

### Participants

There were 572 participants who attempted to take the survey. Participants were screened out of the study if they were not parents or did not answer all the nine attention items correctly. Question examples included “Who was the first president of the United States?,” “Who invented the lightbulb?,” and “What is the current year?” We analyzed a final dataset consisting of 183 valid responses (age: mean 36.9, SD 8.0 years; see Table 1 for more demographic information).

### Study Survey

A common approach to gathering social validity information from stakeholders is to develop short prompts and a questionnaire that can be administered to large groups relatively quickly [15,16]. Participants were directed from their participant recruitment portals to a Qualtrics survey. The full survey is available from the corresponding author.

#### Cell Phone Usage

Fifteen items assessed the participants’ usage of mHealth apps including if they have downloaded apps, how often they use the app, and the platform on which they have downloaded apps. Participants also indicated their willingness to purchase apps and were asked to estimate the amount of money they would spend on a mHealth app. Questions also assessed if participants’ have downloaded an app for their child or adolescent and the focus of the app.

#### Domains of the Theoretical Domains Framework

For the purposes of this study we chose to focus on health behaviors of diet, physical activity, and sleep as these were of primary intellectual interest to us. We selected elements of the TDF that were most directly applicable to mobile phone apps in order to characterize parents’ perception of importance for inclusion in mHealth apps to target diet, physical activity, and sleep. The TDF domain questions were developed in an iterative process by three of the study authors (CC, DF, EB). Each author is a doctoral-level pediatric psychologist with expertise in behavior change. The TDF domain definitions were ranked by each author to evaluate their applicability to mHealth. After the group reached consensus, EB developed examples of mHealth features that would map on to each TDF domain. Examples were refined until consensus agreement was reached among CC, DF, and EB.

Definitions of the TDF domains were provided in the question stem along with examples for how the TDF element would be incorporated into a hypothetical app. Parents rated the helpfulness of that domain for inclusion in an app on a 5-point scale ranging from 1=not helpful to 5=really helpful. Example items included “Apps could also provide a way to assist children and adolescents in scheduling time to practice the behavior. The app could send an alarm for when the child needs to engage in physical activity or send a reminder to help mom and dad cook a nutritious meal, or lastly to lock the phone to encourage not using electronics at night” (example of practice) and “Apps may also be useful in giving the positive benefits of changing these healthy behaviors. For instance, sleep is related to better concentration, diet is related to more energy, and physical activity could lead to weight loss and better sleep” (example of optimism; see Multimedia Appendix 1 for a list of sample survey questions).

All parents were prompted to answer questions on the TDF domains regarding diet, physical activity, and sleep. Attention questions were also interspersed within the study questions as a manipulation check. Question examples include “Who was the first president of the United States?,” “Who invented the lightbulb?,” and “What is the current year?” Incorrect answers to these questions resulted in the participant’s data being excluded from the study.

### Data Analysis

All analyses were conducted using SPSS statistical software, version 23, for frequencies and descriptive statistics. To understand parents’ views on the helpfulness of mHealth apps and the TDF in relation to different health behaviors, one-way between-subjects analyses of variances (ANOVA) were conducted with the construct as the independent variable and type of health behavior (ie, physical activity, diet, and sleep) as the dependent variable. Significant differences between health behaviors were followed up with post hoc comparisons using the Tukey honestly significant difference test to better understand the parents’ ratings of the helpfulness of mHealth apps. Degrees of freedom were larger than the number of participants in the sample because three different ratings (ie, physical activity, diet, and sleep) were provided for each prompt by a single participant. Statistical tests were considered significant if type I error rates were less than 5%.

**Table 1:** Demographics of participants (N=183).

Variable		Participants
Age (years), mean (SD); range		36.9 (8.0); 23-68
**Race, n (%)**		
	Caucasian/White	124 (67.8)
	African American	16 (8.7)
	Asian	28 (15.3)
	Native American	4 (2.2)
	Biracial	6 (3.3)
	Other	5 (2.7)
**Ethnicity, n (%)**		
	Hispanic or Latino	17 (9.3)
	Not Hispanic or Latino	165 (90.2)
	No response	1 (0.0)
**Relationship status, n (%)**		
	Single	31 (16.9)
	Married	126 (68.9)
	Separated	4 (2.2)
	Divorced	22 (12.0)
**Annual family income (US$), n (%)**		
	0-19,999	19 (10.4)
	20,000-39,999	44 (24.0)
	40,000-59,000	38 (20.8)
	60,000-79,999	38 (20.8)
	80,000-99,999	25 (13.7)
	≥100,000	19 (10.4)
**Number of children, n (%)**		
	1	77 (42.1)
	2	69 (37.7)
	3	23 (12.6)
	4-7	14 (7.7)
**Current cell phone platform, n (%)**		
	Android	104 (56.8)
	iPhone	75 (41.0)
	Windows	3 (1.6)
	Blackberry	1 (0.5)

## Results

### Parent mHealth App Use

A total of 105 of 183 parents (57.4%) reported that they had some type of mHealth app on their phone (this could be an app that was preloaded on the phone or downloaded intentionally). The majority of these parents (85/105, 81.0%) reported that they used their mHealth app every day or multiple times a week. However, when asked if they had downloaded a mHealth app, 156 parents (85.2%) reported they had not; additionally, only 29.6% (8/27) of participants who had downloaded a mHealth app reported that they downloaded the app on their child’s cell phone.

### Likelihood of Using mHealth Apps to Target Child Health Behaviors

When asked to rate how much of a problem physical activity was for their child, 70 of 183 (38.3%) parents reported it was little to a lot of a problem; 90 of 183 (49.2%) parents reported diet was a little to a lot of a problem for their child. When asked to rate how much of a problem sleep was for their child, 83 of 183 (45.4%) parents rated sleep as little to a lot of a problem. When asked how likely they would be to use a mHealth app to target their child’s physical activity, most parents (66.7%, 122/183) reported somewhat likely to very likely. A majority of parents (60.6%, 111/183) reported they were somewhat likely to very likely to use a mHealth app to target their child’s diet. Parents also reported that they would be likely to use a mHealth app to target their child’s sleep (51.9%, 95/183 of parents reported somewhat likely to very likely).

### Theoretical Domains Framework and mHealth Behavior Change Techniques

#### Capability

The majority of parents rated the capability component as helpful for their child’s physical activity (73.1%, 132/183 reported somewhat helpful to really helpful), diet (73.8%, 135/183 reported somewhat helpful to really helpful), and sleep (66.7%,122/183 reported somewhat helpful to really helpful). These results suggest that, overall, parents found the mHealth TDF domains related to capability to be helpful strategies for their child’s health behaviors.

Within the knowledge domain, there were no significant differences in parents’ ratings of the helpfulness of the knowledge and knowledge of task environment constructs. This suggests parents may view mHealth apps that provide knowledge and knowledge of task environment to be equally helpful for their child’s physical activity, diet, and sleep behaviors. However, parents rated the helpfulness of procedural knowledge differently depending on the health behavior (*F*_2,545_=5.18, *P*=.006). Post hoc comparisons revealed that parents rated features to enhance physical activity (mean 3.96, SD 1.08, *P*=.008; Cohen *d*=0.31) and diet knowledge (mean 3.91, SD 1.17, *P*=.03; Cohen *d*=0.26) as significantly more helpful than sleep features (mean 3.60, SD 1.21). There was no significant difference between diet and physical activity. Therefore, parents may view mHealth apps that provide training in how to perform the health behavior to be more helpful for their child’s physical activity and diet than sleep.

In the skill domain, there was no significant difference in parents’ ratings of helpfulness of the practice construct or skills construct. Parents may be more likely to view mHealth apps that promote repeated practice for the health behavior or provide education and training on a skill to be helpful for their child’s overall health behaviors. There was a significant difference depending on health behavior when parents rated the helpfulness of skill development (*F*_2,546_=3.57, *P*=.03). Post hoc analyses revealed diet (mean 4.03, SD 1.07, *P*=.02; Cohen *d*=0.26) significantly differed from sleep (mean 3.73, SD 1.21), whereby parents rated features designed to enhance dietary skill as more helpful than sleep skill features. There was no significant difference between physical activity and diet or physical activity and sleep. Therefore, parents may view mHealth apps aimed at skill development to be more helpful for their child’s diet than sleep.

Parents’ ratings of the helpfulness of the decision processes domain did not significantly differ suggesting parents may view mHealth apps that assist with making decisions related to health behaviors to be equally helpful for physical activity, diet, and sleep.

Within the behavioral regulation domain, parents’ ratings did not significantly differ in the action planning and breaking habit constructs. These results suggest parents view mHealth apps that assist with creating a specific plan for healthy behaviors or provide information on how to break bad habits and form healthy habits to be useful for their child’s overall health behaviors. However, there was a significant difference in parents’ ratings of the self-monitoring construct (*F*_2,546_=4.04, *P*=.02). Post hoc analyses revealed parents rated features designed to enhance physical activity self-monitoring (mean 4.11, SD 1.14, *P*=.01; Cohen *d*=0.29) as significantly more helpful than features for sleep self-monitoring (mean 3.78, SD 1.14). There was no significant difference between diet and physical activity or sleep. Therefore, parents may view mobile apps that promote self-monitoring as more helpful for their child’s physical activity than sleep.

#### Motivation

Overall, parents found the use of mHealth apps within the motivation component to be helpful for physical activity (73.2%, 134/183 reported somewhat helpful to really helpful), diet (68.9%, 126/183 reported somewhat helpful to really helpful), and sleep (66.8%, 122/183 reported somewhat helpful to really helpful). Within the motivation component, there was no significant difference in parents’ ratings of the helpfulness in the beliefs about consequences domain, intentions domain, and the optimism domain. These results suggest parents are likely to view mobile apps that remind their child of outcome expectancies and consequences related to health behaviors, understand if their child is ready to make a change, teach their child to think more positively about being healthy, and incorporate their child’s identity in relation to health behaviors as equally helpful toward improving their child’s physical activity, diet, and sleep behaviors.

In the goal domain, there was no significant difference in parents’ ratings of goal setting; however, there was a significant difference in parents’ ratings of distal and proximal goals (*F*_2,545_=5.30, *P*=.005). Post hoc analyses revealed physical activity (mean 4.01, SD 0.96) was significantly different from diet (mean 3.68, SD 1.26, *P*=.02; Cohen *d*=0.29) and sleep (mean 3.67, SD 1.15, *P*=.01; Cohen *d*=0.32). Therefore, parents are likely to view mHealth apps focused on goal setting as equally helpful for their child’s physical activity, diet, and sleep behaviors. However, parents may view mHealth apps that assist with distal and proximal goals to be most helpful for their child’s physical activity.

**Table 2:** Descriptive mean scores^a^ of helpfulness of the mHealth TDF domains.

Domain			Total (N=183), mean (SD)	Child (N=8), mean (SD)	Self (N=19), mean (SD)	None (N=156), mean (SD)
**Capability**						
	**Knowledge**					
		Knowledge	3.55 (1.22)	3.96 (0.96)	4.33 (0.93)	3.43 (1.22)
		Procedural knowledge	3.82 (1.16)	3.92 (1.02)	4.39 (1.02)	3.75 (1.17)
		Knowledge of task environment	3.65 (1.19)	4.17 (0.87)	4.26 (1.03)	3.54 (1.19)
	**Skill**					
		Skill development	3.90 (1.12)	4.21 (0.88)	4.47 (0.78)	3.81 (1.14)
		Practice	3.91 (1.10)	4.17 (0.96)	4.33 (0.85)	3.84 (1.12)
		Skills	3.85 (1.10)	4.25 (0.90)	4.37 (0.84)	3.76 (1.11)
	**Memory, attention, and decision processes**					
		Decision making	3.73 (1.17)	3.96 (1.30)	4.21 (1.10)	3.66 (1.16)
	**Behavioral regulation**					
		Self-monitor	3.94 (1.15)	3.96 (0.86)	4.25 (0.99)	3.90 (1.18)
		Action planning	3.83 (1.16)	4.04 (0.91)	4.25 (0.85)	3.77 (1.19)
		Break habit	3.61 (1.25)	4.16 (0.69)	4.04 (1.04)	3.55 (1.26)
**Motivation**						
	**Beliefs about consequences**					
		Outcome expectancies	3.72 (1.15)	4.04 (1.00)	4.28 (0.98)	3.63 (1.16)
		Consequences	3.66 (1.21)	4.00 (1.14)	4.09 (1.11)	3.59 (1.22)
	**Goals**					
		Goal setting	3.87 (1.05)	3.92 (0.88)	4.39 (.077)	3.81 (1.07)
		Distal and proximal goals	3.79 (1.14)	4.13 (0.80)	3.98 (1.10)	3.75 (1.15)
	**Intentions**					
		Transtheoretical	3.73 (1.17)	4.21 (0.83)	4.21 (1.10)	3.65 (1.18)
	**Optimism**					
		Optimism	3.88 (1.13)	4.21 (1.06)	4.33 (0.89)	3.81 (1.15)
		Identity	3.19 (1.28)	3.33 (1.47)	3.84 (1.10)	3.11 (1.28)
	**Reinforcement**					
		Rewards	3.98 (1.14)	3.79 (1.14)	4.09 (1.11)	3.98 (1.15)
		Reinforcement	3.89 (1.13)	4.17 (0.76)	4.23 (0.98)	3.84 (1.16)
		Incentives	3.90 (1.17)	4.04 (1.12)	3.93 (1.21)	3.89 (1.16)
**Opportunity**						
	**Environmental context**					
		Resources	3.60 (1.23)	3.83 (1.17)	4.12 (1.12)	3.52 (1.23)
		Barriers and facilitators	3.38 (1.24)	4.00 (0.66)	3.93 (1.12)	3.28 (1.25)
	**Social influences**					
		Social comparisons	3.47 (1.32)	3.71 (1.16)	3.84 (1.36)	3.41 (1.32)
		Social support	3.54 (1.24)	3.71 (0.96)	3.84 (1.31)	3.49 (1.25)

^a^Scores were based on a five-point Likert scale (higher scores indicating more helpfulness). Columns are broken down based on participant experience. Self: indicates the parent downloaded a health app; child: indicates that the health app is on the child’s phone; none: indicates that the parent believes that neither they nor the child have downloaded a health app.

**Table 3:** Parents’ ratings of helpfulness of overall TDF by health behavior.

Domain	Physical activity, mean (SD)	Diet, mean (SD)	Sleep, mean (SD)
Capability	3.81 (0.88)	3.86 (0.93)	3.68 (0.98)
Motivational	3.83 (0.87)	3.75 (0.89)	3.69 (0.92)
Opportunity	3.56 (0.97)	3.52 (1.02)	3.41 (1.04)

Within the reinforcement domain, there was no significant difference in parents’ ratings of rewards and reinforcement, suggesting parents viewed mHealth apps that provide rewards for engaging in one of the behaviors or provide encouragement as equally helpful for their child’s physical activity, diet, and sleep. There was a significant difference in parents’ ratings of incentives (*F*_2,546_=4.34, *P*=.01). Parents rated physical activity (mean 4.10, SD 1.12, *P*=.01; Cohen *d*=0.31) significantly greater than sleep (mean 3.75, SD 1.15); this suggests parents may view mobile apps that provide a reward for completing a task of a health behavior to be helpful for physical activity.

#### Opportunity

Overall, parents rated the opportunity component as helpful for physical activity (63.3%,116/183 reported “somewhat helpful” to “really helpful”), diet (60.1%, 111/183 reported “somewhat helpful” to “really helpful”), and sleep (57.4%, 105/183 reported “somewhat helpful” to “really helpful”). There was no significant difference in parents’ ratings of environmental context and resources domain. These results suggest parents are likely to view mHealth apps that provide information on resources related to health behaviors or provide information on barriers or facilitators to be equally helpful for their child’s physical activity, diet, and sleep.

Within the social influences domain, there was no significant difference in parents’ ratings of the social comparison construct. Therefore, parents are likely to view mHealth apps that encourage social comparison or social support to be helpful for their child’s physical activity, diet, and sleep. There was no significant difference between physical activity and diet or sleep. Parents may view mHealth apps that allow friends, family, and health care providers to give encouragement and support to be slightly more helpful for their child’s diet than sleep. Table 2 provides parents’ overall mean ratings by theoretical domains for general health behaviors.

To determine whether there were differences in parental preferences for the capability, opportunity, and motivation domains, we conducted one-way ANOVAs with post hoc *t* tests. In each case, the only significant difference was that parents viewed the opportunity domain as less helpful than either the capability or motivation domains. Table 3 provides parents’ overall mean ratings of the helpfulness of general theoretical domains by physical activity, diet, and sleep.

## Discussion

Results from this study indicate that fewer than one-fifth of parents have downloaded an app on their child’s cell phone to help manage their health behavior. Despite current low adoption, approximately two-thirds of parents indicated a willingness to use a mobile app to help manage their child’s diet and physical activity, whereas approximately half of all parents reported they would use an app to manage their child’s sleep. Within the TDF domain of capability, parents were most interested in increasing their child’s capability as it related to physical activity and diet relative to sleep. However, these differences were relatively small (ie, less than one-third of a standard deviation) suggesting relatively strong interest from parents for using mobile apps to improve their child’s capability to perform all three health behaviors. Within the domain of motivation, parents were again generally interested in using mHealth apps to improve their child’s motivation for healthy behavior. The most notable difference among the health behaviors was for goal setting; findings suggested that parents may most readily associate goal setting with physical activity. Comparing the domains of motivation and opportunity, parents were less enthusiastic about using mHealth apps to improve the opportunity for their children to engage in health behavior across all three health behaviors assessed.

It is noteworthy that parents generally reported lower preference for TDF strategies targeting their child’s sleep. This is an area where one can imagine some discrepancy between the views of a behavioral scientist and a parent. For instance, the behavioral scientist may be interested in teaching a child about sleep hygiene using a mHealth app. However, parents’ responses suggest that they would be less amenable to using an app for this purpose. One explanation may be that parents are not as informed about good sleep practices. It is common for laypeople to have suboptimal knowledge about healthy sleep practices, and to experience poorer sleep as a consequence [17]. In this case, it might be useful to not only work with parents to determine their preference for specific behavior change strategies, but also to educate potential users on good sleep hygiene before asking their opinions on how to change these behaviors. Based on our findings, recent calls for app developers and sleep experts to develop evidence-based guidelines for sleep apps should be expanded to include stakeholders because these groups are likely to see the function of apps through different lenses [18].

Parents were more enthusiastic about using mHealth apps to assist their children with behavioral regulation, with particular enthusiasm shown for physical activity behavior. Strategies such as self-monitoring and goal-setting skills for physical activity were among the most highly preferred for parents. These features are often well-integrated as activity reminders or prompts to set goals in existing mHealth platforms [5]. Parents were less enthusiastic about leveraging social influences to motivate their child’s activity. These findings are consistent with previous work in young adults that found participants are likely to judge self-regulation behavior change techniques as central to physical activity app efficacy, while relatively devaluing the importance of social features [19].

Despite our efforts to recruit a nationally representative sample using Amazon MTurk, our study overrepresents Native Americans and Asians and underrepresents African Americans relative to the 2015 US Census. This is a limiting factor for the current findings because there is well-documented disadvantage conferred on ethnic minority groups in terms of both disproportionate risk and lack of available interventions [20,21]. mHealth approaches can be an equalizing force because of the high adoption of mobile phone technology in minority groups [22]; however, representative samples from stakeholder groups are a necessary first step toward building an equitable intervention framework. Our sample is diverse, but not fully representative. Moreover, there is the potential for selection bias as our sample is only representative of high-performing workers on the MTurk platform who are also parents. Our study did not consider developmental age or cognitive ability as a driver of parent preferences.

Our study did not explicitly assess important factors such as usability. It is relatively well-established that usability modifies the degree to which a user perceives a given behavior change technique as central to the efficacy of an app [23,24]. Consequentially, our study is likely biased upward because respondents are likely assuming that apps are highly usable and that they would engage with them regularly. Our study did not involve stakeholders in the development of our survey. It is important to note that this report is not a definitive assessment of parent preferences. It is one low-cost approach to incorporating an end user and theory into the mHealth app development lifecycle.

It is well documented that evidence-based digital health solutions are not typically disseminated widely after validation. Perhaps even more concerning, the lessons learned from research studies or recommendations provided by expert consensus groups are not typically adopted in commercially available apps [5-7]. It has been argued that there needs to be an interdisciplinary bridge between for-profit technology companies and scientific labs to maximize the uptake of digital health interventions [1]. However, there are many barriers to such collaborations and research labs may need to do some preliminary work before forging a partnership with technologists.

This study presents a low-cost strategy for involving a large number of stakeholders in the discussion of how health behavior theory should be applied in a mHealth intervention. Our approach is innovative in that it took a scientific framework (ie, TDF) and made it digestible to parents so that they could then provide their opinions about features that might appear in a future app. Our survey items discriminated between various health behaviors allowing stakeholders to communicate the different health behaviors that they would like a TDF feature to change. Moreover, we were able to develop a set of consumer opinions about features that were directly linked to elements of the TDF. Similar approaches to app development may help to ensure that stakeholder opinions are included in theoretically sound app development. If successful, it may be possible to develop effective interventions with higher uptake among the target users because theoretically sound features that are rated as desirable by consumers can be included, while undesirable features (even if theoretically sound) could be excluded.

## Appendix

Multimedia Appendix 1 Domain and survey questions.

## Abbreviations

HIT: Human Intelligence Task
mHealth: mobile health
TDF: Theoretical Domains Framework
