# Supplementary material for: Parents’ Perspectives on the Theoretical Domains Framework Elements Needed in a Pediatric Health Behavior App: A Crowdsourced Social Validity Study
Source: JMIR Mhealth Uhealth. 2018 Dec 21;6(12):e192. doi: 10.2196/mhealth.9808 (PMC6320421; doi:10.2196/mhealth.9808)
Supplement: Multimedia Appendix 1 [file mhealth_v6i12e192_app1.docx]

| Domain  Appendix | Survey Question |
| --- | --- |
| Capability  Knowledge  Knowledge  Procedural Knowledge  Knowledge of Task Environment  Skills  Skill Development  Practice  Skills | How helpful do you think it would be for your child to receive education about the health behaviors listed below from a mobile app?  How helpful do you think it would be for your child to receive training in how to perform the health behaviors from a mobile app?  How helpful do you think it would be for a mobile app to teach your child about the people, places, and equipment required to do the behaviors below?  How helpful do you think it would be for your child to have a mobile app that provided training and prompts to practice the behaviors below?  How helpful do you think it would be for your child to have mobile app that promoted repeated practice for the behaviors below?  How helpful do you think it would be for your child to have a mobile app that provided education and training on a new skill for the behaviors below |
| Behavior Regulation  Self-monitoring  Action Planning  Breaking Habit  Memory and Decision Processes  Decision making  Motivation  Beliefs about consequences  Outcome Expectancies  Consequences  Goals  Goal/target setting  Goals (distal/proximal)  Intentions  Transtheoretical  Optimism  Optimism  Identity  Reinforcement  Rewards  Reinforcement  Incentives  Opportunity  Environmental Context and Resources  Resources  Barriers and facilitators  Social Influences  Social comparisons  Social support | How helpful do you think it would be for your child to have a mobile app allow for tracking or recording of the behaviors listed below?  How helpful do you think it would be for your child to have a mobile app assist youth in creating a specific plan for how to be health  How helpful do you think it would be for your child to have a mobile app provide information on how to break bad habits and form healthy habits for the behaviors listed below?  How helpful do you think it would be for your child to have a mobile app assist with making decisions between two or more choices?  How helpful do you think it would be for your child to have a mobile app that provides information on what to expect for each behavior?  How helpful do you think it would be for your child to have a mobile app that provides information and education on the consequences of engaging in the behaviors listed below?  How helpful do you think it would be for your child to have a mobile app provide education and training on setting a goal that is time-specific, can be easily measured in the app, and is realistic for one of the behaviors below?  How helpful do you think it would be for your child to have a mobile app provide information on short-term and long-term goals for the behaviors listed below?  How helpful do you think it would be for your child to have a mobile app that understands if your child is ready to make a change and provide suggestions on how to make that change for the behaviors listed below?  How helpful do you think it would be for your adolescent to have a mobile app that teaches that adolescent how to think more positively about being healthy?  How helpful do you think it would be for your child to have a mobile app that incorporates the youths’ identity and the impact on healthy behaviors?  How helpful do you think it would be for your child to have a mobile app that provides rewards for engaging in one of the behaviors listed below?  How helpful do you think it would be for your child to have a mobile app that provides encouragement to increase their healthy behaviors?  How helpful do you think it would be for your child to have a mobile app provide a reward for completing a task for one of the behaviors below?  How helpful do you think it would be for your child to have a mobile app provide information on things that are needed to be healthy?  How helpful do you think it would be for your child to have a mobile app provide information on the feelings and thoughts that either help or make it difficult to be healthy?  How helpful do you think it would be for your child to have a mobile app provide information on how your child compares to other peers their age on the behaviors below?  How helpful do you think it would be for your child to have a mobile app that allows friends, family, and healthcare providers to give encouragement and support for your child to engage in the behaviors listed below? |
